# In Vitro Evaluation of Dentin Tubule Occlusion for Novel Calcium Lactate Phosphate (CLP) Paste

**DOI:** 10.3390/ma10030228

**Published:** 2017-02-27

**Authors:** Jen-Chang Yang, Hsin-Tai Hu, Sheng-Yang Lee, Sung-Chih Hsieh, Pei-Chi Huang, Chen-Feng Ma, Dian-Yu Ji, Liang-Yu Chang, Nai-Chia Teng

**Affiliations:** 1 Graduate Institute of Nanomedicine and Medical Engineering, College of Biomedical Engineering, Taipei Medical University, Taipei 11052, Taiwan; yang820065@tmu.edu.tw (J.-C.Y.); alex627324@gmail.com (L.-Y.C.); 2 Center for Teeth Bank and Dental Stem Cell Technology, Taipei Medical University, Taipei 11052, Taiwan; seanlee@tmu.edu.tw (S.-Y.L.); supermcf@yahoo.com.tw (C.-F.M.); 3 School of Dentistry, Taipei Medical University, Taipei 11052, Taiwan; hsintaihu1011@hotmail.com (H.-T.H.); endo@tmu.edu.tw (S.-C.H.); alkjjj@gmail.com (D.-Y.J.); 4 Dental Department of Wan-Fang Hospital, Taipei Medical University, Taipei 11052, Taiwan; 5 Graduate Institute of Biomedical Materials and Engineering, Taipei Medical University, Taipei 11052, Taiwan; 6 Dental Department, Taipei Medical University Hospital, Taipei 11052, Taiwan

**Keywords:** dentin hypersensitivity, calcium lactate phosphate (CLP), desensitizing agent

## Abstract

Introduction: The objective of this in vitro study is to evaluate the effective and long-term occlusion of dentinal tubules using a novel calcium lactate phosphate (CLP) based desensitizing agent. Methods: Dentin disks (n = 9) were pre-etched using 1 M lactic acid for 30 s and individually treated with Colgate^®^ Pro-Relief™ paste, CLP paste, and double distilled water (ddH_2_O) by a rubber-cupped handpiece. Dentin disks were analyzed under optical micrographs for pre-treatment, directly after treatment, and 14 days post-treatment. One-way ANOVA and post-hoc Tukey’s test were used to determine whether there were any statistically significant differences in dentinal tubule diameter. Results: A significant decrease occurred in the mean tubule diameter for dentin disks treated with CLP paste. A decrease was observed from 3.52 ± 0.83 µm to 2.62 ± 0.42 µm right after treatment, further decreasing to 1.71 ± 0.45 µm after immersion in artificial saliva for 14 days (*p* < 0.05). Conclusions: The results suggest that the CLP based desensitizing paste has remineralization properties and provides instant and lasting effectiveness in dentinal tubule occlusion.

## 1. Introduction

Dentinal hypersensitivity (DH) has been researched extensively due to its widespread prevalence and is a painful oral health problem that affects many individuals [1]. It is usually characterized as “a short, sharp pain arising from exposed dentin in response to stimuli typically thermal, evaporative, tactile, osmotic or chemical but with a diagnosis of exclusion from any other dental problems” [2]. Etiological factors of DH such as lesion localization and initiation due to abrasion, attrition, erosion, and gingival recession, in tandem with dental procedures contribute to the loss of enamel and cementum, exposing dentinal tubules to the oral environment [3,4]. Odontoblastic transduction theory [5,6], neural theory [7], pain gate control theory [8], and Brannstrom’s hydrodynamic mechanism [9] have been proposed to explain the mechanism of DH. Due to the clinical complexity of DH, effective management strategies have been developed to address its wide array of causal mechanisms.

The dentinal tubule is the gateway through which stimuli gain access to the pulp. The odontoblasts and associated nerve fibers are able to sense the dentinal fluid movement [10]. Thus, treatments to relieve DH are further classified as chemical agents that interrupt the neural response to pain stimuli, or physical agents that occlude the dentin tubules to block the hydrodynamic mechanism by Scherman and Jacobsen [11]. Although there are several commercial desensitizing products that claim to be successful in relieving these symptoms, nowadays sealing the dentinal tubules is considered the favorable way. Treatments such as Nd:YAG laser [12], DP-bioglass paste [13], and tricalcium silicate [14] support the effectiveness of directly accessing dentinal tubules.

Current dentistry developments aim to achieve successful prevention, early intervention, and relatively non-invasive treatments at the first signs of dental disease [15]. The preventive properties of remineralizing agents, typically formulated with calcium and phosphate ions, facilitate ion deposition into voids in demineralized enamel to induce a net mineral gain [16]. To take advantages of remineralizing agents as a desensitizing agent, calcium lactate phosphate (CLP), a soluble calcium salt of calcium oxide, lactic acid, and phospheric acid, was developed in our laboratory. Our aim was to identify whether CLP paste would offer occlusion properties.

## 2. Materials and Methods

### 2.1. Calcium Lactate Phosphate Desensitizing Paste Preparation

Calcium lactate phosphate (CLP), was concocted by loading 3.15 g of lactic acid (88%, MW = 90.08 g/mol, First Chemical, Taipei City, Taiwan), 0.57 g of phosphoric acid (85%, MW = 98.00 g/mol, Sigma-Aldrich, St. Louis, MO, USA), and 1.28 g of calcium oxide (MW = 56.08 g/mol) into a reactor containing 100 mL deionized water for agitating 24 h. After the reaction, the solution was filtered and freeze-dried to harvest CLP in powder form. The resulting product contained 1.00 g of CaCO_3_ (99.9%, FW 100.09, J.T. Baker, KS, USA), 0.06 g of CLP, 0.04 g of hydroxypropyl methyl cellulose (HPMC, 2 wt % in H_2_O, Sigma-Aldrich) and 0.90 g of deionized water.

### 2.2. Pre-Etched Dentin Disk Preparation

**Figure 1:**
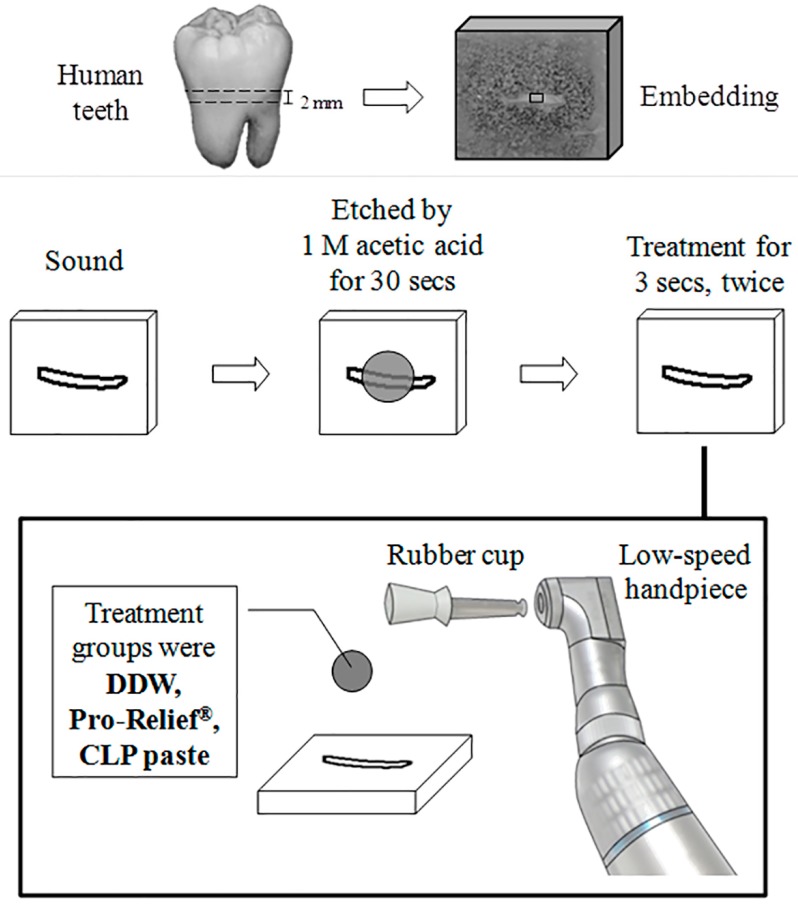
Illustration of the sample preparation and experimental procedure.

Extracted human teeth were collected after informed consent obtained under a protocol approved by the Ethics Committee of the School and Hospital of Stomatology (TMU-JIRB 201104014), Taipei Medical University. The teeth were cleaned thoroughly and stored in normal saline solution (Otsuka, Taiwan) at 4 °C before use. To standardize this evaluation, all the samples were prepared using experimental procedure shown in Figure 1. Dentin discs with a thickness of 2.0 mm approximately, were cut perpendicular to the long axis of the tooth above the cemento-enamel junction (CEJ) by means of a low-speed 0.15 mm diamond saw (Three A Co., Ltd., Taoyuan City, Taiwan). The dentin disc was embedded by acrylic resin (Orthodontic Resin, Hygenic, Milford, DE, USA). After embedding of the specimens, the facial side of each dentin disc was sanded with 600-grit water sandpaper to create a smear layer (1 × 1 mm^2^). The specimens were then polished with 1200-grit water sandpaper. The smear layer was subsequently removed by soaking in 1 M acetic acid solution for 30 s.

### 2.3. Dentinal Tubule Occlusion Test 

Three different treating groups consisting of the aforementioned teeth were evaluated. The groups consisted of teeth treated with Colgate^®^ Sensitive Pro-Relief™ desensitizing paste (Colgate-Palmolive Company, New York, NY, USA), and CLP paste, and a control group treated with ddH_2_O. Polishing rubber cups with dental low-speed handpiece contra angle model was applied at low pressure to the dentine surface at an inclination of about 90° for 3000 rpm. Each group was in contact with the dentin surface at 3 s for two cycles, left to stand for 5 min, and then washed with 10 mL of distilled water.

### 2.4. Optical Microscope Analysis

Treated dentin disks specimens were immersed in artificial saliva and aged in an oven at 37 °C for 14 days [17]. Micrographs of the dentin surface were obtained using an optical microscope (TS100, Nikon, Tokyo, Japan). The mean diameter of dentin tubules was calculated from 20 measurements of each specimen.

### 2.5. Statistical Analysis

The difference in the diameter among the treatment groups and the control group was obtained using one-way analysis of variance (ANOVA) and post-hoc Tukey’s test (a freeware online calculator from http://statistica.mooo.com/), with *p*-values of <0.05 indicating statistical significance.

## 3. Results

The calcium lactate phosphate (CLP) with the chemical formulation of Ca_9_(C_3_H_5_O_3_)_12_(PO_4_)_2_ was prepared by wet process. The measured solubility for CP, CL, and CLP were 0.0, 61.0, and 90.0 g/L water at 25 °C, respectively. The solubility of CLP is higher than the individual component of CP or CL suggesting its amorphous character.

**Figure 2:**
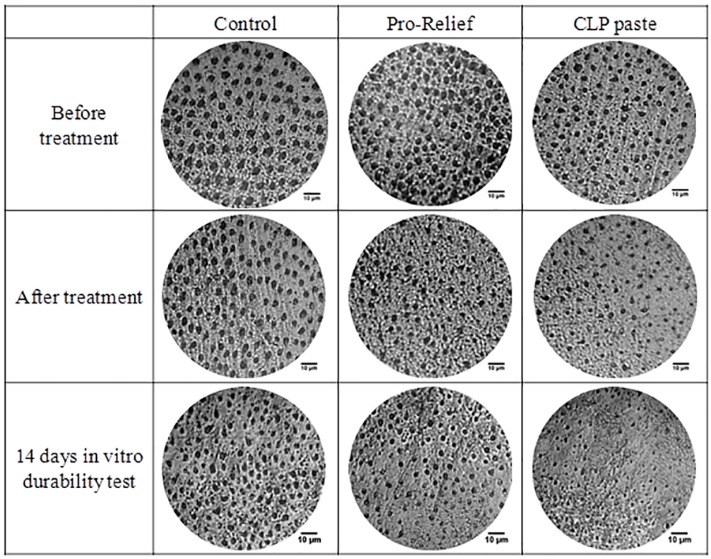
The optical micrographs of occlusal dentin disk surface pre-treatment, immediately post-treatment, and 14 days post-treatment of various desensitizing pastes.

Optical micrographs of the occlusal dentin disk surface pre-treatment, immediately after treatment, and 14 days post-treatment of various desensitizing pastes are displayed in Figure 2. Changes in dentin tube diameter can be observed with visual perception under optical micrographs. Means of dentinal tubule diameter of the dentin disk with various desensitizing pastes and time periods were measured and are summarized in Table 1. By using a CLP paste, the mean dentin tubule diameter was found to significantly decrease from 3.52 ± 0.83 µm to 2.62 ± 0.42 µm right after treatment, further decreasing to 1.71 ± 0.45 µm after immersion in artificial saliva for 14 days (*p* < 0.05). However, Colgate^®^ Sensitive Pro-Relief™ group only showed statistically significant change in dentin tubule diameter between the pre-treatment and 14 days post-treatment phases.

## 4. Discussion

Hypersensitive areas usually coincide with dentinal tubule exposure. The hydrodynamic theory proposes that a stimulus applied on the dentin surface causes movement of tubular fluid and a pressure change across the dentin. This pressure change affects nerves, thus causing pain and discomfort. In fluid dynamics, the Hagen-Poiseuille law correlates with the volume flow rate Q of a Newtonian fluid with the pressure drop ΔP causing by the flow through a long cylindrical pipe with length L under laminar flow condition using $Q=\frac{\pi (\Delta P)D^{4}}{128\mu L}$ [18].

There are various treatment modalities to manage dentin hypersensitivity. Dentinal tubule occlusion or coagulation inside tubules is a physiological way to treat dentin hypersensitivity, which prevents stimuli and dentinal fluid movement [19]. Landry and Voyer believe that there is no ideal desensitizing agent [20]. Grossman (1934) proposed that any treatment for dentin hypersensitivity should be effective by only one application and should satisfy the following parameters: not irritating pulp, not causing pain, easy application, long-lasting effect, not discoloring or staining teeth, not irritating soft tissues or periodontal ligament, and low-cast [21].

There is evidence that depositing a cover layer or inducing in situ formation of natural minerals on dentin surface, in addition to deep penetration into dentinal tubules, provide instant and lasting relief when administered with desensitizing agents [22]. Dentin tubule blocking agents reduce dentin permeability such as potassium oxalate, bioglass, calcium phosphates, arginine-calcium carbonate or dental adhesive materials [13,23]. A reduction in the flow rate reflects the effect of dentinal tubule occlusion was reported by Kim [24]. As such, both Pro-Relief™ and CLP were expected to demonstrate the effect of treating DH to a certain degree based on the results that all the desensitizing agents occluded dentinal tubules. 

Products utilizing calcium phosphate technology are often administered and recommended to treat both demineralization and dentinal hypersensitivity [25,26]. The rationale behind its development was to provide the same minerals found in hydroxyapatite to speed up remineralization in the presence of fluoride. The concept is that by rapidly depositing additional mineral onto the tooth, surface defects would remineralize. It has been extrapolated that these same minerals would also block exposed dentinal tubules and improves dentinal hypersensitivity. However, the relationship between intratubular sealing depth and long-lasting protection when comparing different desensitizing agents is complex due to the variation of deposit quality and material characteristic solubility. Ca(OH)_2_, calcium oxalate, calcium carbonate and calcium fluoride have solubility product constants (Ksp) that vary from 10^−6^ to 10^−10^. In contrast, the Ksp values of most calcium phosphate salts range from 10^−25^ to 10^−50^. CLP is a soluble salt of calcium, lactic acid and phosphoric acid. Its chemical formula was not confirmed. The calcium lactate phosphate (CLP) offers high solubility up to 11.1 g/100 mL water. Furthermore, it dissociates into calcium and phosphate ions in aqueous solutions, and then the ions serve as the building elements for further remineralization. The classical model of crystal formation begins with crystal nucleation, followed by crystal growth. They possess carboxylic acid and phosphate functional groups that act as preferential sites for Ca/P nucleation and subsequent apatite crystallization.

The extent of hydroxyapatite dissolves in a given solution is governed by thermodynamic ion activity product (IAP = (Ca^2+^)_10_(PO4^3−^)_6_(OH^−^)_2_). The requirement for remineralization to occur is when the IAP in the remineralizing solution is less than its product of solubility, Ksp [23]. The increase of the calcium and phosphate ion concentration in oral fluid drives the remineralization process. Thus, the extension of DH protection period is possible when the existing hydroxyapatite crystals on dentin surface acts as nuclei, then accelerates the precipitation of calcium phosphate and causing the further mineralized layer to grow.

Our results showed that the calculated mean ratio of flow rate was down to 31% ((2.62/3.52)^4^ × 100%) right after treatment, and further decreased to 6% ((1.71/3.52)^4^ × 100%) after immersion in artificial saliva for 14 days using CLP pastes. The further reduction in dentinal tubule diameter after incubation in artificial saliva can be attributed to the remineralization process. The high calcium availability provided by CLP, a novel remineralization agent, was attributed to its high solubility and calcium stabilization by random formation of metastable complexes of calcium ions with lactate and phosphate ions. Currently, remineralizing agents like NovaMin™ (bioactive glass containing calcium sodium phosphosilicate) [27,28], Recaldent™ (CPP-ACP casein phosphopeptide-amorphous calcium phosphate) [27,29], Enamelon^®^ (containing stannous fluoride along with calcium and phosphate ions) [30] were utilized as desensitizing agents. Analogous mechanism to these remineralizing agents is expected to provide the same relief to DH as CLP pastes.

Pro-Argin containing 8% arginine–calcium carbonate is a proven in-office desensitizing paste that is effective in providing significant immediate reduction in dentine hypersensitivity for periodontitis patients [31]. However, the Colgate^®^ Sensitive Pro-Relief™ group showed no significant difference for the group right after treatment and immersion in artificial saliva for 14 days. Unlike typical remineralizing agents, Pro-Argin releases no phosphate ions. In addition, the relatively high solubility (0.013 g/L water) of calcium carbonate might offset the net mineral gain [32]. 

Currently, many commercial products claim to offer instant DH relief. The current standards for long-term evaluation when comparing various desensitizing agents are still questionable. The dynamic oral environment is affected by the localized activities of brushing, chewing, and saliva. Consequently, the longevity of DH protection can only be elucidated by conducting long-term in vivo studies.

## 5. Conclusions

The results of the present study, confirmed by SEM analysis of fractured dentin samples, demonstrated that using CLP pastes as desensitizing agents offers good prospects for instant and 14 days constant-increasing dentinal tubule occlusion. The newly developed CLP paste may be a good alternative treatment for dentin hypersensitivity relief. Further research is required to provide evidence of the durability of occlusion of these desensitizing agents under simulated clinical conditions and evidence based correlations between pain and discomfort assessments by a visual analog scale are to be implemented in the future.

## Figures and Tables

**Table 1:** Means of dentinal tubule diameter with various treatments and time periods.

Groups	The Diameter of Dentinal Tubule (μm)		
	ddH_2_O	Pro-Relief Paste	CLP Paste
Before treatment	4.00 ± 0.64 ^a^	3.85 ± 0.49 ^a^	3.52 ± 0.83 ^a^
After treatment	3.13 ± 0.45 ^b^	3.33 ± 0.72 ^a,b^	2.62 ± 0.42 ^c^
In vitro 14 days aging test	3.43 ± 0.87 ^a,b^	3.02 ± 0.56 ^b^	1.71 ± 0.45 ^d^

The data of twenty tubules (*n* = 20) were collected, calculated, and expressed as “mean ± standard deviation”. The data with the same letter tag indicate no statistical difference (*p* > 0.05, one-way ANOVA with post-hoc Tukey HSD test).

## References

[1] Kanapka J.A. (1982). Current treatment for dentinal hypersensitivity. A new agent. Compend. Contin. Educ. Dent..

[2] Canadian Advisory Board on Dentin Hypersensitivity (2003). Consensus-based recommendations for the diagnosis and management of dentin hypersensitivity. J. Can. Dent. Assoc..

[3] Pamir T., Ozyazici M., Baloglu E., Onal B. (2005). The efficacy of three desensitizing agents in treatment of dentine hypersensitivity. J. Clin. Pharm. Ther..

[4] Parolia A., Kundabala M., Mohan M. (2011). Management of dentinal hypersensitivity: A review. J. Calif. Dent. Assoc..

[5] McCormack K., Davies R. (1996). The enigma of potassium ion in the management of dentine hypersensitivity: Is nitric oxide the elusive second messenger?. Pain.

[6] Egbuniwe O., Grover S., Duggal A.K., Mavroudis A., Yazdi M., Renton T., Di Silvio L., Grant A.D. (2014). TRPA1 and TRPV4 activation in human odontoblasts stimulates ATP release. J. Dent. Res..

[7] Lilja J., Nordenvall K.J., Branstrom M. (1982). Dentin sensitivity, odontoblasts and nerves under desiccated or infected experimental cavities. A clinical, light microscopic and ultrastructural investigation. Swed. Dent. J..

[8] Seltzer S. (1978). Pain Control in Dentistry: Diagnosis and Management.

[9] Brannstrom M. (1963). Dentin sensitivity and aspiration of odontoblasts. J. Am. Dent. Assoc..

[10] Bergenholtz G., Horsted-Bindslev P., Reit C. (2010). Textbook of Endodontology.

[11] Scherman A., Jacobsen P.L. (1992). Managing Dentin Hypersensitivity: What Treatment to Recommend to Patients. J. Am. Dent. Assoc..

[12] Liu H.C., Lin C.P., Lan W.H. (1997). Sealing depth of Nd:YAG laser on human dentinal tubules. J. Endod..

[13] Kuo T.C., Lee B.S., Kang S.H., Lin F.H., Lin C.P. (2007). Cytotoxicity of DP-bioglass paste used for treatment of dentin hypersensitivity. J. Endod..

[14] Dong Z., Chang J., Deng Y., Joiner A. (2011). Tricalcium silicate induced mineralization for occlusion of dentinal tubules. Aust. Dent. J..

[15] Limeback H. (2012). Comprehensive Preventive Dentistry.

[16] Cochrane N.J., Cai F., Huq N.L., Burrow M.F., Reynolds E.C. (2010). New approaches to enhanced remineralization of tooth enamel. J. Dent. Res..

[17] Amaral F.L., Colucci V., Palma-Dibb R.G., Corona S.A. (2007). Assessment of in vitro methods used to promote adhesive interface degradation: A critical review. J. Esthet. Restor. Dent..

[18] Bird R.B., Stewart W.E., Lightfoot E.N. (2002). Transport Phenomena.

[19] Miglani S., Aggarwal V., Ahuja B. (2010). Dentin hypersensitivity: Recent trends in management. J. Conserv. Dent..

[20] Landry R.G., Voyer R. (1990). Treatment of dentin hypersensitivity: A retrospective and comparative study of two therapeutic approaches. J. Can. Dent. Assoc..

[21] Grossman L.E. (1935). The treatment of hypersensitive dentine. J. Am. Dent. Assoc..

[22] Chiang Y.C., Chen H.J., Liu H.C., Kang S.H., Lee B.S., Lin F.H., Lin H.P., Lin C.P. (2010). A novel mesoporous biomaterial for treating dentin hypersensitivity. J. Dent. Res..

[23] Naveena P., Nagarathana C., Sakunthala B.K. (2014). Remineralizing Agent—Then and Now—An Update. Dentistry.

[24] Kim S.Y., Kim E.J., Kim D.S., Lee I.B. (2013). The evaluation of dentinal tubule occlusion by desensitizing agents: A real-time measurement of dentinal fluid flow rate and scanning electron microscopy. Oper. Dent..

[25] Tung M.S., Eichmiller F.C. (1999). Dental applications of amorphous calcium phosphates. J. Clin. Dent..

[26] Wolff M.S. (2009). Dentin hypersensitivity, the biofilm, and remineralization: What is the connection?. Adv. Dent. Res..

[27] Yang H., Pei D., Chen Z., Lei J., Zhou L., Huang C. (2014). Effects of the application sequence of calcium-containing desensitising pastes during etch-and-rinse adhesive restoration. J. Dent..

[28] Shivaprasad B.M., Padmavati P., Sanghani N.N. (2014). Chair Side Application of NovaMin for the Treatment of Dentinal Hypersensitivity—A Novel Technique. J. Clin. Diagn. Res..

[29] Madhavan S., Nayak M., Shenoy A., Shetty R., Prasad K. (2012). Dentinal hypersensitivity: A comparative clinical evaluation of CPP-ACP F, sodium fluoride, propolis, and placebo. J. Conserv. Dent..

[30] Kaufman H.W., Wolff M.S., Winston A.E., Triol C.W. (1999). Clinical evaluation of the effect of a remineralizing toothpaste on dentinal sensitivity. J. Clin. Dent..

[31] Pepelassi E., Rahiotis C., Peponi E., Kakaboura A., Vrotsos I. (2015). Effectiveness of an in-office arginine-calcium carbonate paste on dentine hypersensitivity in periodontitis patients: A double-blind, randomized controlled trial. J. Clin. Periodontol..

[32] Tegethoff F.W., Rohleder J., Kroker E. (2001). Calcium Carbonate: From the Cretaceous Period into the 21st Century.

